# On the Galvanic Electricity

**Published:** 1800-08

**Authors:** 


### *On the GALVANIC ELECTRICITY.*

SINCE the account of the extenſion of Galvani's experiments, given at one of Sir Joſeph Banks's meetings, and ſoon after by Dr. Garnet at the Royal Inſtitution, we have been very deſirous of preſenting our readers with an accurate ſtatement of what is now known on that curious and intereſting ſubject. Mr. Nicholſon's Philoſophical Journal for July, furniſhes us with the moſt authentic means.

The 9th article of that number is intitled, "*Account of the new Electrical or Galvanic Apparatus of Sig.* ALEX. VOLTA, *and Experiments performed with the ſame.*

"From motives of delicacy," ſays Mr. Nicholſon, "to the inventor of the moſt curious and important combination hereafter to be deſcribed, I forbore giving an account of its conſtruction and effects in the laſt number of this Journal, though it has now been a ſubject of great attention among philoſophers for near two months. It appeared proper to avoid the publication of facts, originally flowing from the liberal communication of the worthy preſident of the Royal Society, until the paper of the inventor had been read to that learned body; and this could not be done till very lately, becauſe the latter part of his memoir did not arrive till long after the firſt four pages.

"The Right Honourable Sir Joſeph Banks, Bart. P. R. S. having favoured my friend Anthony Carliſle, Eſq. with the peruſal and conſideration of theſe four pages at the latter end of laſt April, I had the pleaſure to look them over with him, immediately after which he conſtructed an inſtrument according to Sig. Volta's directions. The experiments made with this this will form part of the prefent communication; but in the firft place, I fhall endeavour to relate the leading particulars of the communication made to the Royal Society, which no doubt will hereafter appear at large in their Tranfactions.

" The portion of letter which firft arrived from Sig. Volta, is dated from Como in the Milanefe, March 20, 1800. This, together with the fubfequent parts, contains a detailed account of the inftrument, of which the following is one of the moft convenient forms.

" Take any number of plates of copper, or, which is better, of filver, and an equal number of tin, or which is much better, zinc, and a like number of difcs, or pieces of card, or leather, or cloth, or any porous fubftance capable of retaining moifture. Let thefe laft be foaked in pure water, or which is better, falt and water, or alkaline lees. The filver or copper may be pieces of money. Build up a pile of thefe pieces; namely, a piece of filver, a piece of zinc, and a piece of wet card: then another piece of filver, a piece of zinc, and a piece of wet card: and fo forth, in the fame order (or any other order, provided the pieces fucceed each other in their turn) till the whole number intended to be made ufe of is builded up. The inftrument is then completed.

" In this ftate it will afford a perpetual current of electricity, through any conductor communicating between its upper and lower plates; and if this conductor be an animal, it will receive an electrical fhock as often as the touch is made, by which the circuit is completed. Thus, if one hand be applied to the lower plate, and the other to the upper, the operator will receive a fhock, and that as often as he pleafes to lift his finger and put it down again.

" This fhock refembles the weak charge of a battery of immenfe furface, and its intenfity is fo low, that it cannot make its way through the dry fkin. It is, therefore, neceffary that a large furface of each hand fhould be well wetted, and a piece of metal be grafped in each, in order to make the touch, or elfe that the two extremities of the pile fhould communicate with feparate veffels of water, in which the hands may be plunged.

" The commotion is ftronger the more numerous the pieces. Twenty pieces will give a fhock in the arms, if the above precautions be attended to. One hundred pieces may be felt to the fhoulders. The current of electricity acts on the animal fyftem while the circuit is complete, as well as during the inftant of commotion, and the action is abominably painful at any place where the fkin is broken.

" That the energy of the apparatus is the effect of an electric tric ſtream or current, is proved by the condenſer with which Sig. V. aſcertained the kind of the electricity and obtained its ſpark. He finds the action ſtrongeſt, or moſt pungent, on wounds on the minus ſide of the apparatus, or where the wounds give out electricity, a fact alſo obſervable in the common electric ſpark.

" The theory of the learned inventor, if I rightly apprehend him, is, that it is a property of ſuch bodies as differ in their power of conducting electricity, that when they are brought into contact they will occaſion a ſtream of the electric matter. So that if zinc and ſilver be made to communicate immediately by contact, there will be a place of good conducting energy; and if they be made to communicate mediately by means of water, there will be a place of inferior conducting energy: and wherever this happens there will be a ſtream or current produced in the general ſtock of electricity. This is not deduced as the conſequence of other more ſimple facts; but is laid down as a general or ſimple principle grounded on the phenomena.

" As the current of electricity will be reſiſted by the different conductors, he remarks that the metals may touch in a ſingle point, or be ſoldered together; but that the humid ſurfaces muſt be more extended.

" By many experiments, he finds that the conſequences are the ſame, whether the zinc and ſilver touch each other, or whether the communication be made by ſeveral different metals, provided the water be in contact with the zinc and the ſilver only.

" Where zinc is uſed, ſalt water is preferable to alkaline lees, but the contrary when tin is made uſe of inſtead of the zinc.

" The effect is much increaſed by elevation of temperature.

" He was ſurpriſed to find that the Galvanic flaſh of light was no greater with this apparatus than with a pair of plates; but is was produced when the conductor of the circuit was applied to any part of the face, or even to the breaſt. The ſtrongeſt action was when the touching plate was held between the teeth, ſo as to lie upon the tongue. In this caſe the lips and tongue were convulſed, the flaſh appeared before the eyes, and the taſte was perceived in the mouth.

" Two blunt probes were inſerted in the ears, and the ſhock paſſed through the head, after which the communication was kept up. A peculiar ſound, like crackling or boiling, was heard; but the author did not think it prudent to make this experiment repeatedly.

" The senſe of ſmell could not be excited, beeauſe, as Sig. V. remarks, this electricity cannot be made to diffuſe itſelf in the air.

" As the diſcs become dry, and loſe their power, Sig. V. endeavoured to prevent this effect by incloſing the column in wax or pitch, and in this he has ſo far ſucceeded, that he has fitted up two colums of twenty pieces each, which have acted well for ſome weeks, and he hopes will for months.

" The combination, which he thinks the moſt inſtructive, conſiſts of a row of glaſſes or cups (not of metal) containing warm water or brine. Into each of theſe is plunged a plate of zinc and another of ſilver, not touching each other. From theſe plates reſpectively proceed tails or prolongations, which communicate with or touch the plates of the outer glaſſes in ſuch a manner, that the zinc of the firſt cup communicates with the ſilver of the ſecond; the zinc of the ſecond with the ſilver of the third; the zinc of the third, &c. progreſſively and regularly through the whole row. The communication between the firſt and laſt glaſſes gives the ſhock, &c. The plates in the fluid are directed to be about an inch ſquare; but the contacts above the water may be as ſmall as the operator pleaſes.

" Sig. Volta makes honourable mention of my conjectural theory of the torpedo. After remarking that my inductions were the moſt probable that the exiſting theory of electricity could at that time afford, he proceeds to make various objections, needleſs to be here detailed, and then offers his own new and ſtriking apparatus as more nearly reſembling the torpedinal organ. I need not anticipate the reader in the happy points of reſemblance between their ſtructure and effects.

" Thus far," ſays Mr. Nicholſon, " I have followed this able philoſopher; who, to his former reſearches into the nature and laws of electricity, has now added a diſcovery which muſt for ever remove the doubt whether Galvaniſm be an electrical phenomenon. But I cannot here look back without ſome ſurprize, and obſerve that the chemical phenomena of Galvaniſm, which had been ſo much inſiſted on by Fabbroni, more eſpecially the rapid oxydation of the zinc, ſhould conſtitute no part of his numerous obſervations.

" On the 30th of April, Mr. Carliſle had provided a pile conſiſting of ſeventeen half crowns, with a like number of pieces of zinc, and of paſteboard, ſoaked in ſalt water. Theſe were arranged in the order of ſilver, zinc card, &c. which order I ſhall denote by ſaying, that the ſilver was undermoſt, that is to ſay, under the zinc; and I make this remark becauſe ſome philoſophers have uſed the expreſſion that the ſilver was undermoſt undermoſt when they uſed the order of ſilver, card zinc, &c. which, as the reader will eaſily perceive, is contrary to the order here ſpoken of. This is of no conſequence to the effect, though it is material to a clear underſtanding of the terms we uſe. This pile gave us the ſhock as before deſcribed, and a very acute ſenſation wherever the ſkin was broken. Our firſt reſearch was directed to aſcertain that the ſhock we felt was really an electrical phenomenon. For this purpoſe the pile was placed upon Bennett's gold leaf electrometer, and a wire was then made to communicate from the top of the pile to the metallic ſtand or foot of the inſtrument. So that the circuit of the ſhock would have been through the leaves, if they had diverged. But no ſigns of electricity appeared. Recourſe was then had to the revolving doubler, deſcribed at page 95 of our preſent volume. The plate A was connected with the top of the electrometer and the ſilver end of the pile; and the plate B and ball were made to touch the top of the ſyſtem by an uninſulated braſs wire. The doubler had been previouſly cleared of electricity by twenty turns in connection with the earth. The negative divergence was produced in the electrometer. Repeated experiments of this kind ſhewed that the ſilver end was in the minus, and the zinc end in the plus ſtate.

"In all theſe experiments it was obſerved, that the action of the inſtrument was freely tranſmitted through the uſual conductors of electricity, but ſtopped by glaſs and other non-conductors. Very early in this courſe, the contacts being made ſure by placing a drop of water upon the upper plate, Mr. Carliſle obſerved a diſengagement of gas round the touching wire. This gas, though very minute in quantity, evidently ſeemed to me to have the ſmell afforded by hydrogen when the wire of communication was ſteel. This, with ſome other facts, led me to propoſe to break the circuit by the ſubſtitution of a tube of water between two wires. On the 2d of May we, therefore, inſerted a braſs wire through each of two corks inſerted in a glaſs tube of half an inch internal diameter. The tube was filled with New River water, and the diſtance between the points of the wires in the water was one inch and three quarters. This compound diſcharger was applied ſo that the external ends of its wire were in contact with the two extreme plates of a pile of thirty-ſix half crowns with the correſpondent pieces of zinc and paſteboard. A fine ſtream of minute bubbles immediately began to flow from the point of the lower wire in the tube, which communicated with the ſilver, and the oppoſite point of the upper wire became tarniſhed, firſt deep orange, and then black. On reverſing the tube, the gas came from the other point, which was now loweft, while the upper in its turn became tarnifhed and black. Reverfing the tube again, the phenomena again changed their order. In this ftate the whole was left for two hours and a half. The upper wire gradually emitted whitifh filmy clouds, which, towards the end of the procefs, became of a pea green colour, and hung in perpendicular threads from the extreme half inch of the wire, the water being rendered femiopaque by what fell off, and in a great part lay, of a pale green, on the lower furface of the tube, which, in this difpofition of the apparatus, was inclined about forty degrees to the horizon. The lower wire of three quarters of an inch long, conftantly emitted gas, except when another circuit, or complete wire, was applied to the apparatus; during which time the emiffion of gas was fufpended. When this laft mentioned wire was removed, the gas re-appeared as before, not inftantly, but after the lapfe of four beats of a half fecond clock ftanding in the room. The product of gas, during the whole two hours and a half, was two-thirtieths of a cubic inch. It was then mixed with an equal quantity of common air, and exploded by the application of a lighted waxed thread.

" It might feem almoft unneceffary to have reverfed the order of the pile in building up, as reverfing the tube muft have anfwered exactly the fame purpofe. We chofe, however, to do this, and found that when the zinc was at the bottom, its effects were reverfed, that is to fay, the gas ftill came from the wire communicating with the filver, &c.

" We had been led by our reafoning on the firft appearance of hydrogen to expect a decompofition of the water; but it was with no little furprize that we found the hydrogen extricated at the contact with one wire, while the oxygen fixed itfelf in combination with the other wire at the diftance of almoft two inches. This new fact ftill remains to be explained, and feems to point at fome general law of the agency of electricity in chemical operations. As the diftance between the wires formed a ftriking feature in this refult, it became defirable to afcertain whether it would take place to greater diftances. When a tube three-quarters of an inch in diameter, and thirty-fix inches long, was made ufe of, the effect failed, though the very fame wires, inferted into a fhorter tube, operated very brifkly. The folicitation of other objects of enquiry prevented trial being made of all the various intermediate diftances; but from the general tenor of experiments, it appears to be eftablifhed, that this decompofition is more effectual the lefs the diftance between the wires, but that it ceafes altogether when the wires come into contact.

" May 6.—Mr. Carlifle repeated the experiment with copper per wires and tincture of litmus. The oxydating wire, namely, from the zinc side, was the lowest in the tube; it changed the tincture red in about ten minutes as high as the upper extremity of the wire. The other portion remained blue. Hence it seems either an acid was formed, or that a portion of the oxygen combined with the litmus, so as to produce the effect of an acid.

" It may be here offered as a general remark, that the electric pile with card, or with woollen cloth, continues in order for about two days, or scarcely three; that from a series of glasses set up by Mr. Carlisle, as well as from the pile itself, it appears that the same process of decomposition of water is carried on between each pair of plates, the zinc being oxyded on the wet face, and hydrogen given out; that the common salt is decomposed, and exhibits an efflorescence of soda round the edges of the pile, extruded, most probably, by the hydrogen: and that on account of the corrosion of the faces of the zinc, it is necessary to renew them previous to each construction of the pile. This may be done by scraping or grinding. I found it most convenient to lay the piece in a hole in a board, and give it a stroke with a float file, or file of which the teeth are not crossed. It might, perhaps, be less troublesome to clean them with diluted muriatic acid; but this I have not tried.

" As the ample field of physiological research to which Mr. Carlisle's attention is directed, and the multiplicity of my own avocations, rendered it less convenient for us to pursue our enquiries together, I constructed an apparatus for my own use. Zinc was laminated to the twenty-fourth part of an inch in thickness, and pure silver to the one-thousandth part of an inch, that is to say, as thin as our flatting mills can bring it.

" Of these metals I made two sets, namely, sixteen pieces of silver of two inches in diameter, and sixteen pieces of 1.8 inch diameter, with their correspondent plates of zinc and wetted card. The small pile was first prepared, and whether it were that these thin pieces were more disposed to admit the water between the metallic faces of contact, or from whatever other cause it may have arisen, it did not appear by any experiment, that the whole set, though so greatly exceeding the pile of half-crowns in surface, was capable of doing more in the decomposition of water, or in communicating the shock. But this, with other facts, seems to shew, that the repetition of the series is of more consequence to this action, than the enlargement of surface; and also that the thickness of the plates, though it may be attended with convenience, most probably affords no addition to the force. I must also add, that I have no reason to recommend my pile, though at first sight it seemed to to poſſeſs cheapneſs and convenience. The plates of zinc are too thin to bear frequent cleaning or renewing after corroſion of the ſurface, and the ſilver, though it is ſcarcely acted on in this ſituation, is too thin to be conveniently wiped or handled.

“ The ſpontaneous electricity of the doubler preſented an objection to the ſtrict fidelity of its reſults; whence I thought it deſirable to give my pile a trial with the condenſer. The foot or ſtand of my electrometer is a braſs plate truly flat, and 3.8 inches in diameter. A piece of thin Perſian ſilk was tied ſmoothly upon the face of this plate, and it was then placed upon another braſs plate, upon which it was moved about horizontally, in order to accumulate electricity by friction; the electrometer itſelf being uſed as the handle by graſping the top. It was found that this treatment produced very weak ſigns of electricity when the electrometer was lifted up. The lower braſs plate was then placed on the top of the ſmall pile, and the condenſing electrometer placed upon it. A communication was then made, by means of a wire from the lower or ſilver end of the pile to the upper plate of the condenſer, or foot of the electrometer. In this ſituation it is evident, that the charge of the pile was employed in producing oppoſite ſtates of electricity in the condenſor, which would be ſhewn when the plates came to be ſeparated. The wire of communication being taken away, the electrometer was lifted, and the leaves diverged and ſtruck. It became neceſſary, therefore, to repeat the experiment, taking care to lift the electrometer more gradually. The divergence took place as before, and it was increaſed by preſenting excited ſealing wax towards the bottom of the electrometer. And as the top of the pile had by compenſation diminiſhed the ſame divergence, it is clear that the electricity of the top of the pile, *viz.* of the zinc, was contrary to that of ſealing wax; that is to ſay, the zinc was in the plus ſtate. After a number of repetitions of this experiment with the ſame invariable reſult, the pile was then carefully overſet, without diſturbing the relative arrangement of its parts; ſo that the zinc was now at the bottom, and the ſilver at the top. The electricity of the ſilver was then tried a number of times, by preciſely the ſame proceſs as before, and it exhibited an equal degree of intenſity, but it was minus or negative. In one of theſe experiments, I certainly ſaw the ſpark at the time of completing the circuit, and afterwards with the ſame pile, when I was expreſſly looking for it. But it is leſs neceſſary to dwell on theſe facts, as the ſtronger combinations have exhibited this effect with much greater perſpicuity.

“ The decompoſition of water, and oxydation of metallic wire, wire, gave birth to a variety of ſpeculations and projects of experiments. Among others it became a queſtion, what would be the habitude of metals of difficult oxydation. Two wires of platina, one of which was round, and one-fortieth of an inch in diameter, and the other nearly of the ſame maſs, but flatted to the breadth of one twenty-fifth of an inch, were inſerted into a ſhort tube of a quarter of an inch inſide diameter. When placed in the circuit, the ſilver ſide gave a plentiful ſtream of fine bubbles, and the zinc ſide alſo a ſtream leſs plentiful. No turbidneſs nor oxydation, nor tarniſh appeared, during the courſe of four hours continuance of this operation. It was natural to conjecture, that the larger ſtream from the ſilver ſide was hydrogen, and the ſmaller oxygen. Thick gold leaf was tried with the ſame effects. A wire of braſs was then ſubſtituted inſtead of one of the ſlips of gold. When the braſs was on the minus, or ſilver ſide, the two gaſes were extricated for two hours, without oxydation as before; but when the braſs was, by reverſing the tube, brought to the plus ſide, it became oxyded in the ſame manner as if both the wires had been braſs. When the ſlips of gold were long ſubjected to this action, the extremity of the ſlip communicating with the zinc, acquired a coppery or purpleiſh tinge, which was deepeſt near the end. Whether this aroſe from oxydation of the gold, or of the copper, of which gold leaf contains about a ſeventieth part, cannot from this experiment be decided.

"The ſimple decompoſition of water by platina wires without oxydation, offered a means of obtaining the gaſes ſeparate from each other. With this intention, Mr. Carliſle's pile of thirty-ſix was combined with my two ſets of ſixteen repetitions. His pile was built with the zinc uppermoſt, and mine in the reverſe order; ſo that by connecting the upper plates the whole conſtituted one range, and the communications could be made from the bottom of the one to the bottom of the other. The two platina wires were made to protrude out of two ſeparate tubes, each containing a little water, and through the oppoſite corks of each were paſſed copper wires of communication. Theſe tubes were ſlightly greaſed on the outſide to prevent their becoming damp; and in this ſtate the extremities, armed with the platina, were plunged in a ſhallow glaſs veſſel of water, in which two ſmall inverted veſſels, quite full of water, were ſo diſpoſed, that the platina of one tube was beneath one veſſel, and the platina of the other tube was beneath the other, the diſtance between their extremities being about two inches. The copper wires of theſe tubes reſpectively were made to communicate with the extremities of the intire pile of ſixty-eight ſets. A cloud of gas aroſe from each wire, but but moft from the filver, or minus fide. Bubbles were extricated from all parts of the water, and adhered to the whole internal furface of the veffels. The procefs was continued for thirteen hours, after which the wires were difengaged, and the gafes decanted into feparate bottles. On meafuring the quantities, which was done by weighing the bottles, it was found, that the quantities of water difplaced by the gafes, were refpectively, 72 grains by the gas from the zinc fide, and 142 grains by the gas from the filver fide; fo that the whole volume of gas was 1.17 cubic inches, or near an inch and a quarter. Thefe are nearly the proportions in bulk of what are ftated to be the component parts of water. The gas from the zinc fide, being tried with one meafure of nitrous gas, contracted to 1.25, and did not contract more by the addition of another meafure; the gas from the filver fide, by the fame treatment, contracted to 1.6. The air of the room, on trial, contracted to 1.28. From the fmallnefs of the quantity no attempt was made to detonate the air from the zinc fide, but a portion of that from the filver fide being mixed with one-third of atmofpheric air, gave a loud detonation.

" Upon the above it may be remarked, that it does not feem probable that oxygen was afforded by both wires, but that they were mixed by the circumftances of the experiment. For the gafes being extricated in extremely minute bubbles beneath the inverted veffels, caufed a flow afcending current confifting of water mixed with thofe bubbles, many of which were undoubtedly too fmall to be difcerned. This afcending current gave out as much of its gas at the top of the veffel, as had time to conglomerate; but the extremely minute bubbles would return in the defcending current, and be repeatedly carried up before this effect could take place. Such a continual circulation, or ftream, the lower part of which paffed down into the faucer, muft at length have occafioned the whole mafs of fluid to become replete with thefe minute bubbles, which would break at the open furface, and be loft, or attach themfelves to the fides of the veffel, as was feen to be the cafe. What proportion may have thus difappeared is uncertain; but it is highly probable, that one confequence of the imperfections of our apparatus, was to occafion both the inverted veffels to become receptacles for the gafes from both wires indifcriminately; though moft plentifully in each, from the wire immediately beneath its mouth. If this reafoning may be admitted for the prefent, till the experiment is repeated in clofed veffels, it will be fair to reckon the whole diminution on both the quantities. The whole diminution was 1.15, whence it would follow, follow, that the purity of the oxygen eſtimated in Prieſtley's manner, would be expreſſed by the number 0.85.

"On account of the length of this communication, I ſhall at preſent forbear to enter into any conſiderations of theory, but ſhall conclude with a conciſe mention of the effects of a pile of one hundred half crowns, and a chemical incident, which appears to be the moſt remarkable of thoſe which I have yet obſerved.

"The pile was ſet up with pieces of green woollen cloth ſoaked in ſalt water. It gave ſevere ſhocks, which were felt as high as the ſhoulders. The tranſition was much leſs forcible through a number of perſons, but it was very perceptible through nine. The ſpark was frequently viſible when the diſcharge was made in the dark, and a gleam of light was alſo, in ſome inſtances, ſeen about the middle of the column at the inſtant of the exploſion. The aſſiſtants were of opinion that they heard the ſpark.

"The extrication of the gaſes was rapid and plentiful by means of this apparatus. When copper wires were uſed for the broken circuit, with muriatic acid diluted with 100 parts of water in the tube, no gas, nor the leaſt circulation of the fluid was perceived, when the diſtance of the wires was two inches. A ſhort tube, with two copper wires very near each other in common water, was made part of the circuit, and ſhewed by the uſual phænomena, that the ſtream of electricity was rapidly paſſing. The wires in the muriatic acid were then ſlided within a third of an inch of each other. For the ſake of brevity, I avoid enumerating the effects which took place during ſeveral hours, and ſimply ſtate; that the minus wire gave out ſome hydrogen during an hour, while the plus wire was corroded, and exhibited no oxyde; but a depoſition of copper was formed round the minus, or lower wire, which began at its lower end: that no gas whatever appeared in this tube during two hours, though the depoſition was going on, and the ſmall tube ſhewed the continuance of the electric ſtream; and that the depoſition at the end of four hours formed a ramified metallic vegetation, nine or ten times the bulk of the wire it ſurrounded.

"In this experiment it appeared, that the influence of electricity increaſing the oxydability of the upper wire, and affording naſcent hydrogen from the lower, cauſed the latter to act as the precipitant of a ſolution of one and the ſame metal.

"We are in want of a meaſure of the intenſity of the action of theſe machines. Will this be derived from the quantities of water decompoſed, or of gas extricated under like circumſtances in given times? Or from any change of temperature? Or what other commensurate incident?—Mr. Carlisle has not found that the water in the tube, while under this agency, did produce the slightest effect on a very small and delicate thermometer."

The next article contains, "*Some Experiments and Observations on Galvanic Electricity. By Mr.* W. CRUICKSHANK, *Woolwich. Communicated by the Author.*

"In subjecting a number of fluids to the action of Galvanism, several facts have been discovered, which to me, at least, are perfectly new, and which appear to throw some light on the nature and powers of this new influence.

"I shall, therefore, without any further apology, give a brief detail of some of the most important, hoping that they may prove acceptable to those who are employed in the same pursuits.

"I shall not give any particular account of the apparatus employed, being a pile, and not differing materially from that in use. I shall only just observe, that it consisted of plates of zinc and silver, of about 1.6 inches square, and that the number of each employed in the following experiments varied from 40 to 100, according to the power required.

"I found that a solution of the muriate of ammonia answered better for moistening the interposed papers than common water.

"When the machine was in full action, sparks, which were perfectly visible in the day time, could be taken at pleasure, by making a communication in the usual way between the extremities of the pile, and a small report or snap could be heard; the shock given at that time was very strong, and a gold leaf electrometer, placed in the circle of communication, was very sensibly affected: these circumstances, some of which I believe have been already ascertained by Messrs. Nicholson and Carlisle, shew the strong resemblance of this influence to electricity. These gentlemen have likewise discovered that Galvanism decomposes water with much greater facility than electricity, but with phenomena somewhat different."

Mr. Cruickskank then enters upon an account of his curious experiments; for which, on account of the length of this article, we must refer our readers to Mr. Nicholson's Journal.

